# Risk factors for delirium in acutely admitted elderly patients: a prospective cohort study

**DOI:** 10.1186/1471-2318-5-6

**Published:** 2005-04-13

**Authors:** Johanna C Korevaar, Barbara C van Munster, Sophia E de Rooij

**Affiliations:** 1 Department of Clinical Epidemiology and Biostatistics, Academic Medical Centre, Univ. of Amsterdam, The Netherlands; 2 Department of Internal Medicine, Academic Medical Centre, Univ. of Amsterdam, The Netherlands

## Abstract

**Background:**

Delirium is a neuropsychiatric syndrome frequently observed in elderly hospitalised patients and can be found in any medical condition. Due to the severe consequences, early recognition of delirium is important in order to start treatment in time. Despite the high incidence rate, the occurrence of delirium is not always identified as such. Knowledge of potential risk factors is important. The aim of the current study is to determine factors associated with the occurrence of a prevalent delirium among elderly patients acutely admitted to an internal medicine ward.

**Methods:**

All consecutive patients of 65 years and over acutely admitted to the Department of Internal Medicine of the Academic Medical Centre, Amsterdam, a university hospital, were asked to participate. The presence of delirium was determined within 48 hrs after admission by an experienced geriatrician.

**Results:**

In total, 126 patients were included, 29% had a prevalent delirium after acute admission. Compared to patients without delirium, patients with delirium were older, more often were cognitively and physically impaired, more often were admitted due to water and electrolyte disturbances, and were less often admitted due to malignancy or gastrointestinal bleeding. Independent risk factors for having a prevalent delirium after acute admission were premorbid cognitive impairment, functional impairment, an elevated urea nitrogen level, and the number of leucocytes.

**Conclusions:**

In this study, the most important independent risk factors for a prevalent delirium after acute admission were cognitive and physical impairment, and a high serum urea nitrogen concentration. These observations might contribute to an earlier identification and treatment of delirium in acutely admitted elderly patients.

## Background

Delirium is a complex neuropsychiatric syndrome with an acute onset, characterized by disturbances of consciousness, attention, cognition, and perception. Delirium can be found in any medical condition and is the most common reason for acute cognitive dysfunction in hospitalised elderly patients [1-3]. This syndrome occurs in about 10 to 25% of all acute admissions to a general hospital. The frequency in older patients is higher, 20% to 40% [4]. Due to the aging of the population, the absolute number may increase in the future.

Delirium has been associated with a poor outcome: prolonged hospitalisation, decreased cognitive and physical functioning, increased nursing home admission, and with a threefold higher morbidity and mortality risk [5,6]. Due to the severe consequences, recognition of delirium is important to start treatment in time. Despite the high incidence rate, the occurrence of delirium is not always identified. Several studies reported that between 32% and 67% of delirious patients were not recognized by their physicians [7,8]. There are a number of hypotheses for this lack of recognition. First, delirium is not always regarded as an important clinical syndrome, because it is varied and the multiple aetiology defies the classic disease model to look for a single cause of disease. In addition, delirium is often believed to present with agitation, hallucination, and inappropriate behaviour, whereas it also often presents with lethargy and decreased activity. Finally, the fluctuating course of the delirium may confound the diagnosis [9].

The patho-physiology of delirium is still poorly understood, although a number of mechanisms have been hypothesized. Delirium might be the result of changes in neurotransmitter systems, in cytokines, and in the lipid metabolism [10-16]. Delirium and Alzheimer's disease (AD) may share several patho-physiological features as they share several symptoms [17]. Moreover, preliminary results support the assumption that genetic variation plays a role [18].

Several studies have examined risk factors that might predispose, or influence the development of delirium. A systematic review identified 27 articles studying 61 different risk factors [19]. Results of these 27 studies were not conclusive. Most studies found an increased risk for delirium in dementia, medical illness, and alcohol abuse. For risk factors like medication, male gender, or serum concentrations, of e.g. urea, creatinine, sodium, potassium or glucose, some studies did find an increased risk for delirium, whereas other studies did not find an increased risk of these factors [20-23]. Finally, most of these studies determined risk factor for new-onset delirium in hospitalised patients [24,25]. Yet, prevalent delirium can be a major problem as well, especially among acutely admitted patients. A prompt recognition of the syndrome is important to initiate appropriate treatment as soon as possible. Therefore, more knowledge of potential risk factors for acutely admitted patients is important.

The aim of the current study is to determine factors associated with a prevalent delirium among acutely admitted elderly patients to an internal medicine ward.

## Methods

### Patients

All consecutive patients of 65 years and over acutely admitted to the Department of Internal Medicine of the Academic Medical Centre, Amsterdam, a university teaching hospital, were invited. Patients admitted to our hospital are sometimes referred because of the university function, but about 60% are directly admitted to our hospital. Patients were excluded from the study if they were unable to speak or understand Dutch or English, if they or their relatives did not give permission for the study, if they came from or were transferred to another ward than Internal Medicine, or left the ward within 48 hours. Due to logistic limitations, a random sample of included patients was taken for the current study regarding risk factors and genetic variation. More detailed information regarding medication and DNA was collected of these selected patients. Inclusion period was between January 2003 and February 2004. Before enrolment, informed consent was obtained from the patient or substitute decision-maker. The hospital's Medical Ethics Committee approved the study.

### Procedures

Members of the team completed an initial multidisciplinary evaluation for all study participants within 48 hrs after admission. The team was composed of a geriatric physician, a fellow in geriatric medicine, and two research nurses trained in geriatric medicine. Demographic and clinical data were collected. Severity and number of comorbidities were scored with the Charlson comorbidity index [26]. The final score was divided in 3 categories; mild (0 or 1 point), moderate (2 or 3 points) and severe (more than 3 points). Biochemistry values, i.e. urea nitrogen, creatinine, glucose, haemoglobin, natrium, and potassium concentrations, and leucocytes count were obtained from a serum blood taken within 48 hrs after admission.

Within 48 hrs after admission, the research nurses interviewed patients, medical and nursing staff. Cognitive impairment was recorded by two validated instruments (MMSE, IQCODE) at the time of hospital admission. The MMSE (Mini Mental State Examination) is the most widely used screening instrument for detection of cognitive impairment in the elderly [27]. Many studies have shown that the MMSE has got a high construct validity and test-retest reliability [28]. The MMSE measures cognitive functioning on a scale of 0 (poor) to 30 (excellent), with a score less than 24 indicating cognitive impairment. The IQCODE (Informant Questionnaire on COgnitive DEcline) assesses the possible presence of dementia before admission based on the response of an informant who had known the patient for at least ten years and could assess any decline in memory or cognition [29]. The informant was asked to recollect the situation 2 weeks before admission and to compare it with the situation 10 years before. The score is an average of the 16-item scores, each rated from 1 (much improved) to 5 (much worse). Patients with a mean score of 3.9 or more were considered to have dementia [30]. Final classification for having cognitive impairment was based on the MMSE score for patients without delirium, whereas for patients with delirium, the combination of both instruments (MMSE and IQCODE) was applied. In case of conflicting outcome, the score of the IQCODE was used.

The geriatric physician or the fellow scored the presence of delirium within 48 hrs after admission with the CAM (Confusion Assessment Method). The CAM is a structured interview of delirium symptoms based on the Diagnostic and Statistical Manual of Mental Disorders criteria (DSM-III-R). This instrument have been found reliable, sensitive and specific [31]. The delirium could have been present at admission, or developed within 48 hrs after admission.

The KATZ ADL scale is a 15-item scale for measuring functional status in a geriatric population. The KATZ-ADL consists of one scale for patients and one for their relative or informant [32]. KATZ-ADL as scored by the informant of a patient was taken as the final ADL score. In case this score was missing, the KATZ-ADL score of the patient was taken. Once more, the informant was asked to recall the situation 2 weeks before admission. The nurses that collected the risk scores were blinded to the presence or absence of delirium in the patient.

All medication before hospital admission was registered. The number of prescribed drugs was scored. Psychopharmaca included benzodiazepines, antidepressive medication such as selective serotonin reuptake inhibitors and tricyclic antidepressants, and antipsychotic medication.

Based on the literature and our clinical experience, we predefined five different types of drugs as suspected of being a high-risk drug for obtaining delirium [33-35]; namely anticholinergic medication, benzodiazepines, narcotic analgetics, corticosteroids and antihistaminics. Cholinergic drugs were classified according to the list of Han *et al.*[35], all drugs scored with 3 points were considered cholinergic drugs. We completed this list with parasympathicolytics. Benzodiazepines included sedative-hypnotics and anxiolytics. The antihistamincs group contains only H1 receptor blocking agents.

### Statistical analysis

Standard descriptive statistics were used. Univariate and multivariate logistic regression analysis was performed to identify risk factors of delirium. All variables with a p-value <0.20 in the univariate analysis were used in the multivariate model. A backward elimination procedure was used to define the final risk factors. All variables with a p-value >0.10 were eliminated from the model. To confirm the final risk factors, the multiple logistic regression analysis was repeated with a forward selection procedure (all variables with a p-value <0.10 were included).

## Results

During the inclusion period 576 patients were admitted to the internal ward. Of these patients, 88 patients came from another ward, resulting in 488 eligible patients. 182 patients were not included because no informed consent was given or they were unable to speak or understand Dutch or English. In total, 306 patients were included, a random sample of 126 patients was selected for the current study. Non-selected and selected patients were similar regarding mean age, the percentages male, and the frequency of patients with a prevalent delirium. Mean age of the non-selected patients was 78.1 (SD: 8.5), 45% were male and 28% of these patients had a prevalent delirium after acute hospital admission. For the 126 selected patients mean age was 79.1 (7.8), 41% were male and 36 patients (29%) had a prevalent delirium after acute hospital admission.

Baseline characteristics of the 126 selected patients with and without a prevalent delirium are presented in Table 1. Patients with delirium were significantly older, had more often cognitive impairment, and were more impaired in daily activities compared to patients without delirium. Reason for admission was less frequently a malignancy, or a gastrointestinal bleeding, and more frequently water or electrolyte disturbances for patients with delirium. On average, delirious patients had a significantly higher level of serum urea nitrogen at admission.

**Table 1:** Baseline characteristics of acutely admitted elderly patients with and without a prevalent delirium after acute admission.

	Patients with delirium	Patients without delirium
Number	36 (29%)	90 (71%)
Age (yrs.) *	82.1 (7.2)	77.8 (7.8)
65–70 (%)	8%	17%
70–75 (%)	14%	29%
75–80 (%)	14%	14%
80–85 (%)	25%	18%
≥ 85 (%)	39%	22%
Male (%)	50%	38%
Education (yrs.)	8.9 (2.1)	8.8 (3.2)
Comorbidity (%)		
Mild	25%	19%
Moderate	31%	31%
Severe	44%	50%
Admission reason (%) *		
Infectious disease	53%	56%
Malignancy	6%	17%
Gastrointestinal bleeding	3%	11%
Water and electrolyte disturbances	19%	2%
Other	19%	14%
MMSE: Cognitive impairment (%) *	89%	41%
IQCODE: Cognitive impairment (%) *	89%	24%
Katz ADL (%) *		
0	0%	10%
1–3	9%	37%
4–6	15%	19%
≥ 7	77%	35%
Biochemistry		
Urea nitrogen (mmol/L) *	15.9 (13.6)	10.6 (6.2)
Creatinine (μmol/L)	175 (223)	137 (193)
Glucose (mmol/L)	8.5 (5.1)	8.5 (5.3)
Haemoglobin (mmol/L)	7.6 (1.5)	7.2 (1.6)
Sodium (mmol/L)	134.6 (8.5)	133.9 (5.6)
Potassium (mmol/L)	4.1 (0.8)	4.0 (0.7)
Leucocytes (× 10^9^/L)	11.2 (5.8)	13.5 (7.2)
CRP (mg/L)	119.3 (113.6)	99.0 (93.5)

Mean values (SD) are given for continuous variables

* p-value <0.05: patients with delirium versus without prevalent delirium after acute admission.

Table 2 presents the type of medication prescribed to more than 20% of the patients. The majority of the most frequently prescribed drugs are similar for patients with and without delirium, except for psychopharmaca. This type of drugs is prescribed to 31% of the patients with delirium, whereas 17% of the patients without delirium were taking these drugs. Patients without a prevalent delirium were on nearly 5 different prescribed drugs before admission, whereas delirious patients were taking on average 4.4 different drugs before admission. This difference was not statistically significant.

**Table 2:** Medication prescribed before admission to more than 20% of the acutely admitted elderly patients with and without a prevalent delirium after acute admission; descending order. Number of patients (percentages) are given per medication type.

Patients with delirium < 48 hours	Number (%)	Patients without delirium	Number (%)
Glucose lowering medication	14 (39%)	Diuretics	40 (44%)
Antithrombotics	13 (36%)	Antithrombotics	28 (31%)
Gastrointestinal medication	13 (36%)	Analgetics	27 (30%)
Diuretics	12 (33%)	Antihypertensives	27 (30%)
Psychopharmaca	11 (31%)	Glucose lowering medication	27 (30%)
Sympathicolytics	10 (28%)	Gastrointestinal medication	27 (30%)
Analgetics	9 (25%)	Sympathicolytics	26 (29%)
Spasmolytics and vasodilators	9 (25%)	Spasmolytics and vasodilators	19 (21%)
Antihypertensives	8 (22%)		
			
Number of drugs used before admission (mean (SD))	4.4 (3.2)		4.9 (3.6)

Information on the number of patients taking one or more of the predefined high-risk medications is presented in Table 3. Delirious patients obtained more often drugs suspected of being a drug with a high risk, especially benzodiazepines, 17% versus 10%, and narcotic analgetics (14% versus 7%). Yet these differences were not significant.

**Table 3:** List of drugs suspected of being a high-risk drug for a delirium, presented for patients with and without a prevalent delirium after acute admission.

	Patients with delirium	Patients without delirium
	(N = 36)	(N = 90)
Suspected high-risk medication before admission (% patients)		
Benzodiazepines (%)	17%	10%
Narcotic analgetics (%)	14%	7%
Corticosteroids (%)	14%	10%
Antihistaminics (%)	3%	2%
Cholinergic drugs (%)	9%	7%
Any of these 5 medications (%)	34%	27%

* p-value <0.05: patients with prevalent delirium versus without delirium after acute admission.

Based on univariate logistic regression analysis, risk factors for a prevalent delirium after acute admission are higher age, cognitive impairment, impaired physical functioning, admission reason, and urea nitrogen level (Table 4). No increased risk was observed for mean number of prescribed drugs, or the use of drugs suspected of being a high-risk drug. Results of the multivariate logistics regression analysis are presented in Table 5. Independent predictors for an increased risk for a prevalent delirium after acute admission were cognitive impairment, impaired physical functioning, increased urea nitrogen level, and the number of leucocytes. The backward and the forward selection procedure resulted in the same final risk factors (data not shown).

**Table 4:** Univariate logistic regression analysis of risk factors for having a prevalent delirium after acute admission

Variable	Unadjusted Hazard ratio (95% CI)	p-value
Age (yrs.)	1.08 (1.02–1.13)	<0.01
Male	1.62 (0.74–3.53)	0.23
Comorbidity		
Mild	1.00	
Moderate	0.74 (0.26–2.16)	0.58
Severe	0.68 (0.25–1.81)	0.43
Cognitive impairment	10.98 (3.56–33.83)	<0.01
Katz ADL		
0–4	1.00	
5–6	4.22 (0.90–19.92)	0.07
≥ 7	11.76 (3.23–42.77)	<0.01
Admission reason		
Infectious disease	0.71 (0.25–2.04)	0.52
Malignancy	0.25 (0.04–1.41)	0.12
Gastrointestinal bleeding	0.19 (0.02–1.77)	0.14
Water and electrolyte disturbances	6.50 (1.05–40.13)	0.04
Other	1.00	
Urea (mmol/L)	1.06 (1.01–1.11)	0.01
Creatinine (μmol/L)	1.00 (1.00–1.00)	0.36
Leucocytes (* 10^9^/L)	0.95 (0.88–1.01)	0.11
CRP	1.00 (1.00–1.01)	0.31
Number of medications	0.96 (0.86–1.08)	0.51
Benzodiazepines (%)	1.84 (0.60–5.62)	0.29
Narcotic analgetics (%)	2.31 (0.66–8.1)	0.19
Corticosteroids (%)	1.48 (0.46–4.78)	0.51
Antihistaminics (%)	1.28 (0.11–14.58)	0.84
Cholinergic drugs (%)	1.30 (0.31–5.50)	0.72
Any of these 5 medications (%)	1.41 (0.61–3.27)	0.42

**Table 5:** Multivariate logistic regression of risk factors for having a prevalent delirium after acute admission. Backward selection procedure of all variables with a p-value <0.20 in the univariate regression analysis

Variable	Adjusted Hazard ratio (95% CI)	p-value
Cognitive impaired	9.48 (2.27–39.54)	<0.01
		
Katz ADL		
0–4	1.00	
6–5	8.14 (1.08–61.31)	0.04
≥ 7	14.13 (2.26–88.24)	<0.01

Urea (mmol/L)	1.10 (1.02–1.18)	<0.01
		
Leucocytes (* 10^9^/L)	0.87 (0.79–0.97)	0.01

## Discussion

In this prospective cohort study, nearly 30% of acutely admitted elderly patients had delirium within 48 hrs after admission. Increased risk for developing delirium was found for cognitive and physical impairment, elevated urea nitrogen level, and the number of leucocytes. No increased risk for developing delirium was noticed for comorbidity or type or number of medication.

A possible reason for not finding an increased risk of comorbidity could be the limited number of included patients. The use of a more liberal inclusion criteria in the multivariate logistic regression analysis, elimination of all variables with a p-value >0.15, resulted into the same final model. In the literature, inconclusive results can be found regarding the impact of the severity of the disease on the risk of delirium [20,23-25]. So, the limited number of included patients does not seem to be the sole reason for no association between comorbidity and the risk of delirium. Another possible explanation could be the balancing relationship of comorbidity with cognitive impairment. The more cognitive impaired, the less physical illness might be needed to become delirious.

No effect of type or number of drugs prescribed on the risk of delirium was observed. In the current literature, results on the association between anticholinergic drugs and delirium are conflicting. Both in patients with an incident as in patients with a prevalent delirium, some studies did find an increased risk [33,34,36], whereas other studies did not find an effect of anticholinergic drugs [22,23,37,38]. In addition, it could be possible that not just the medication type itself is associated with the risk of delirium, but rather the dosage of the medication, or a recent change in type or dosage of prescribed medication. We did not collect data on these detailed topics, nor did we collect the compliance of the patient to the prescribed medication.

Most studies report an increased risk associated with older age. A reason for not confirming this finding is the strong correlation between age and impaired physical functioning in our study. Indeed, if we excluded the KATZ ADL score, higher age is an independent risk factor for having delirium within 48 hrs after admission.

Our results could have been obscured due to selectively inclusion of patients, as not all patients gave informed consent. However, we found that nearly 30% of the acutely admitted patients had a prevalent delirium after acute admission. This finding is in concordance with the literature; a frequency between 20% and 40% is usually found. Moreover, the frequency in demented patients was much higher, 46% of these patients had delirium. This finding is supported by the literature as well.

Cognitive impairment was the strongest risk factor for a prevalent delirium; we found a ninefold increased risk. This effect is also reported in other studies for the development of delirium after admission. The amount of reported increased risk varies between 2.8 and 9.0 [20,23,24]. An increased urea nitrogen level was associated with an increased risk of a prevalent delirium. A most likely explanation for this finding is that an increased urea nitrogen concentration is, among others, an indication of dehydration. The effect of dehydration on risk of delirium was found in other studies as well [7,24,39]. Moreover, if we did not take urea nitrogen level in the multivariate analysis, we find a significant increased risk for the admission reason water and electrolyte disturbances (data not shown). Finally, we found that a higher number of leucocytes was associated with a lower risk of delirium. We cannot explain this finding, we speculate that this might be due to the higher percentages of non-delirious patients with infectious diseases and malignancies, both associated with a higher level of leucocytes. Indeed, the highest leucocytes counts were found in the non-delirious patients with reason of admission malignancy or infectious disease.

## Conclusions

In this study the independent risk factors for a prevalent delirium after acute admission are premorbid cognitive and physical impairment, and a high urea nitrogen level. These observations might contribute to an earlier identification and treatment of delirium in acutely admitted elderly patients.

## Competing interest

The author(s) declare that they have no competing interests.

## Authors' contributions

SR is the principal investigator of the study; she designed the protocol, supervised its progress, and was involved in the acquisition of the data. BM was involved in the planning of the study and the acquisition of the data. JK was responsible for the statistical analysis. JK and BM drafted the manuscript. All authors contributed to the interpretation of the data, revisions of the paper, and read and approved the final manuscript.

## Role of the funding source

Funding source was our own hospital, it was an unrestricted grant.

## Pre-publication history

The pre-publication history for this paper can be accessed here:


